# Anti-CXCL8 Autoantibody: A Potential Diagnostic Biomarker for Esophageal Squamous Cell Carcinoma

**DOI:** 10.3390/medicina58101480

**Published:** 2022-10-18

**Authors:** Huili Chen, Guiying Sun, Zhuo Han, Huimin Wang, Jiaxin Li, Hua Ye, Chunhua Song, Jianying Zhang, Peng Wang

**Affiliations:** 1 Department of Epidemiology and Statistics, College of Public Health, Zhengzhou University, Zhengzhou 450001, China; 2 State Key Laboratory of Esophageal Cancer Prevention & Treatment, Zhengzhou University, Zhengzhou 450052, China; 3 Henan Institute of Medical and Pharmaceutical Sciences, Zhengzhou University, Zhengzhou 450052, China

**Keywords:** esophageal squamous cell carcinoma, bioinformatic analysis, anti-cxcl8 autoantibody, biomarker

## Abstract

*Background and Objectives*: Esophageal squamous cell carcinoma (ESCC) is one of the most common malignancies. Anti-tumor associated antigen autoantibodies (TAAbs) can be used as biomarkers for tumor detection. The aim of this study was to identify a reliable TAAb as the diagnostic marker for ESCC. *Materials and Methods*: The Cancer Genome Atlas (TCGA) database was used to screen candidate genes. The mRNA expression of the key gene was then verified by micro array dataset GSE44021 from the Gene Expression Omnibus (GEO) database and the diag nostic value of the corresponding autoantibody to the key gene in ESCC was detected by enzyme-linked im muno sorbent assay (ELISA). *Results*: CXCL8 was identified as the key gene. The dataset GSE44021 showed that CXCL8 mRNA expression was prominently over-expressed in ESCC tissues compared with normal tissues. ELISA results showed that the level of anti-CXCL8 autoantibody in ESCC patients was significantly higher than in normal controls and the receiver operating char ac teristic (ROC) curve indicated that anti-CXCL8 autoantibody could discriminate ESCC patients from normal controls, with the area under the ROC curve (AUC) for the verification cohort, and the validation cohort were 0.713 and 0.751, respectively. *Conclusions*: Our study illustrated that anti-CXCL8 autoantibody had good diagnostic value, and may become a candidate biomarker for ESCC.

## 1. Introduction

Esophageal cancer (EC), an aggressive upper gastrointestinal malignancy, ranks sixth in overall mortality among cancers, with an estimated 544,000 deaths from EC in 2020 [1]. EC patients are usually found in the middle or late stage, since the early symptoms are not obvious [2]. To date, the five-year survival rate for EC is about 15–20% [3], but if EC is detected earlier, the survival rate can reach 85% [4,5]. Endoscopy is currently the main method for EC screening, but its low accuracy, high cost, and invasiveness limit its application in the population [6]. Noticeably, about 90 per cent of EC is esophageal squamous cell carcinoma (ESCC) [7]. Consequently, novel diagnostic methods for ESCC that are easy to accept and can compensate for the deficiency of traditional diagnostic methods are urgently needed. In recent years, biomarker-based disease-detection methods have not only improved accuracy and effectiveness of non-endoscopic screening for ESCC, but are also almost non-invasive [8].

More and more studies have indicated that anti-tumor associated antigen autoantibodies (TAAbs) have existed in serum before clinical manifestation of tumors. In addition, TAAbs have the characteristics of strong persistence, long half-life, and convenient detection. These excellent properties make TAAbs a promising serological biomarker for the diagnosis of ESCC [9,10]. At present, many TAAbs have been used for the diagnosis of ESCC, such as NY-ESO-1, STIP1, MMP7, and Hsp70. However, the sensitivity or specificity of these TAAbs cannot meet the needs of ESCC as biomarkers for clinical diagnosis [11,12]. Therefore, it is important to identify other TAAbs with high specificity and sensitivity for the diagnosis of ESCC.

Nowadays, The Cancer Genome Atlas (TCGA) database and the Gene Expression Omnibus (GEO) database are increasingly recognized by researchers. More and more biomarkers for ESCC were identified based on TCGA and GEO databases. For example, Li et al. identified 14 microRNAs as new biomarkers for ESCC, which were also associated with lymph node invasion and metastasis. Bhushan et al. used the cancer genomic dataset to identify and validate the FGF12 as a biomarker for ESCC [13,14]. These studies provided a new understanding of the development of new diagnostic biomarkers for ESCC.

In this study, TCGA database was utilized to screen the differentially expressed genes (DEGs). The protein–protein interaction (PPI) network was used to identify hub genes, which provided references for the identification of tumor-associated antigens (TAAs) associated with ESCC. CXCL8 was selected for further study, and its mRNA expression level was verified by microarray dataset GSE44021 from the GEO database. The corresponding autoantibody of CXCL8 in human serum samples was detected by ELISA to explore its potential as a diagnostic biomarker for ESCC.

## 2. Materials and Methods

### 2.1. Differential Expression Gene Analysis

In this study, the TCGA data portal was used to obtain gene-expression profiles for EC. With the cutoff criteria |log2 fold change (FC)| > 2 and adjusted *p* < 0.05, the “limma” package in R (version 4.0.3) software (https://www.r-project.org/, accessed on 10 May 2021) was applied to identify DEGs. The DEGs identified from TCGA were shown via volcano plot.

### 2.2. Functional Annotation and Hub Genes Screening

The search tool for the retrieval of interacting genes (STRING) database (https://string-db.org/, accessed on 20 May 2021) (version 11.5) was used for gene ontology (GO) and Kyoto Encyclopedia of Genes and Genomes (KEGG) analyses, additionally, to construct the PPI network. The minimum interaction score for the PPI network was 0.4 [15]. The cytoHubba software (integrated into Cytoscape 3.8.2 software, Bethesda, MD, USA) was used to determine hub genes by using the degree topological algorithm [16]. Among the 10 hub genes we selected, CXCL8, with the highest degree, was deemed to be the most closely relevant to ESCC.

### 2.3. CXCL8 mRNA Expression Validation

In order to verify CXCL8 mRNA expression in ESCC, data on the GSE44021 standardized expression profile containing 146 samples was obtained from the GEO database (https://www.ncbi.nlm.nih.gov/geo/query/acc.cgi, accessed on 22 May 2021) and the GEO2R tool was used to compare the different CXCL8 mRNA expressions of ESCC and normal esophageal tissues.

### 2.4. Study Population

In this study, 210 ESCC primary patients and 210 normal controls were recruited from a third-level grade A hospital in Henan province. In a 1:2 ratio, all participants were randomly assigned to verification set and validation set. All ESCC patients in this study were confirmed by pathological examination; also, none of the patients suffered from autoimmune diseases or inflammatory diseases, or received any treatment until blood samples were collected. Autoimmune diseases, esophagus-related diseases were excluded in the normal controls. This study was approved by the Ethics Committee of Zhengzhou University and all participants provided written informed consent before joining this research. Table 1 showed the detailed clinical information of all participants. Blood samples were separated by centrifugation at 3000 rpm for 5 min and stored at −80 °C until further analysis.

### 2.5. Enzyme-Linked Immunosorbent Assay

The serum anti-CXCL8 autoantibody was detected by indirect ELISA in ESCC patients and normal controls. Purified recombinant protein CXCL8 was purchased from the Cloud-Clone Corporation (Wuhan, China). Indirect ELISA has been described in detail in our previous study [17]. The cutoff value for detecting a positive response was set at the mean optical density (OD) value of control sera plus one standard deviation (mean+ SD).

### 2.6. Statistical Analysis

IBM statistical software (version 25.0) and GraphPad Prism 8.0 were used for statistical analysis and two-sided *p* values < 0.05 were regarded as statistically significant. The differences in expression levels of autoantibodies between ESCC patients and normal controls were compared by using the nonparametric test. Receiver operating characteristic (ROC) curves were used to assess the diagnostic ability of anti-CXCL8 autoantibody. Meanwhile, Youden’s index, likelihood ratio, and predictive value were calculated to evaluate the ability of anti-CXCL8 autoantibody to differentiate ESCC patients from normal controls. The area under the ROC curve (AUC) values of different clinical subgroups were analyzed by DeLong test. The frequency of anti-CXCL8 autoantibody in clinical subgroups was analyzed by chi-square test.

## 3. Results

### 3.1. Identification of DEGs

**Figure 1:**
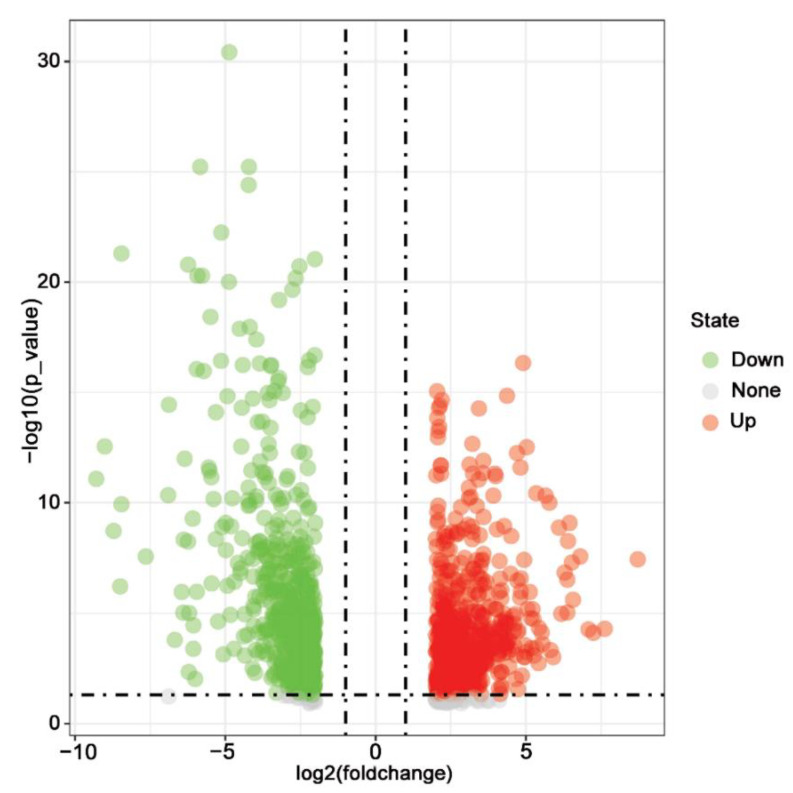
Volcano plot of all DEGs obtained from TCGA database. DEGs were selected with |log FC| > 2 and *p*-value < 0.05. The up-regulated, down-regulated, and unchanged genes were shown in red, green, and grey, respectively.

On the basis of cutoff criteria (|log FC| > 2.0 and *p* < 0.05), 1306 genes were identified as DEGs, including 715 up-regulated DEGs and 591 down-regulated DEGs. The “ggplot2” package of R software was used to draw a volcano plot (Figure 1). Only up-regulated DEGs were chosen for the following analyses in this research.

### 3.2. Functional Annotation and PPI Analysis for the Up-Regulated Genes

**Figure 2:**
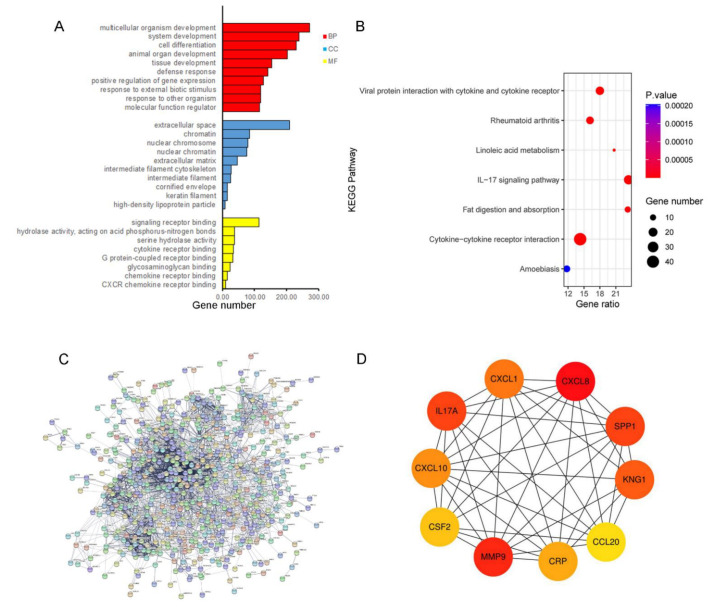
Functional annotation and identification of hub genes via the PPI network. (**A**) Gene ontology enrichment analysis. BP, Biological Process; CC, Cellular Components; and MF, Molecular Function. (**B**) KEGG enrichment analysis. (**C**) PPI network of DEGs analyzed by STRING database. (**D**) Detection of hub genes from the PPI network of common DEGs. The highlighted 10 genes are CXCL8, MMP9, IL17A1, SPP1, KNG1, CXCL1, CXCL10, CRP, CSF2, and CCL20.

The most enriched GO terms in biological process (BP) group, molecular function (MF) group, and cellular component (CC) group were shown in Figure 2A. In terms of BP group, the abundance of DEGs was associated with multicellular organism development, system development, and cell differentiation. For the CC group, the DEGs were remarkably related to extracellular space, chromatin, and nuclear chromosome. The DEGs were mainly related to signaling receptor binding and serine hydrolase activity by MF analysis. KEGG analysis revealed that the cytokine–cytokine receptor interaction, viral protein interaction with cytokine and cytokine receptor, and IL-17 signaling pathway were mainly enriched signaling pathways (Figure 2B). Then, the PPI network was constructed by inputting the up-regulated genes to STRING, including 706 nodes and 2916 edges (Figure 2C). The hub genes were sequenced according to their degree value. The functional roles of identified hub genes were shown in Table 2. Meanwhile, the PPI network of the hub genes was constructed, which had a strong interaction with each other (Figure 2D).

### 3.3. Validation mRNA Expression of CXCL8

**Figure 3:**
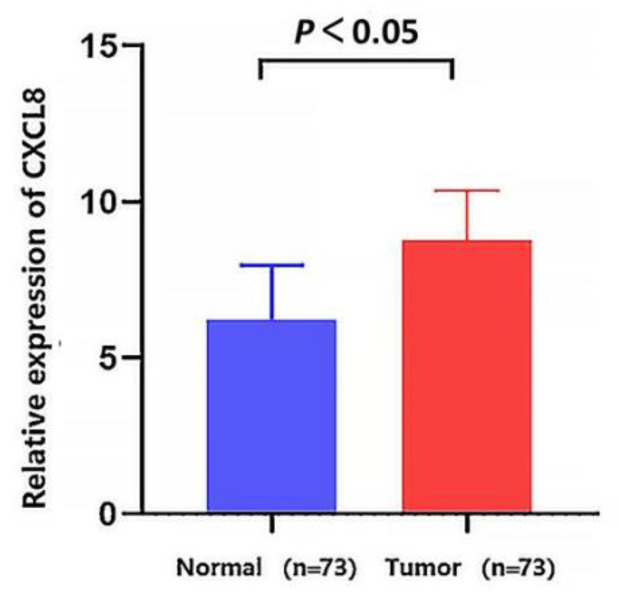
Validation of mRNA expression level of CXCL8 in the GEO database. The mRNA expression level of CXCL8 was up-regulated in ESCC tissues compared with normal tissues (*p* < 0.05). *p* value was calculated using the *t*-test.

Among hub genes, CXCL8 was considered to be the most critical gene in EC with the highest connectivity degree = 86. In the GEO database, the dataset GSE44021 showed that the mRNA expression level of CXCL8 was up-regulated in ESCC tissues compared with normal tissue (Figure 3). Therefore, CXCL8 was confirmed as a candidate TAA for the following study.

### 3.4. Level and Diagnostic Value of Anti-CXCL8 Autoantibody

**Figure 4:**
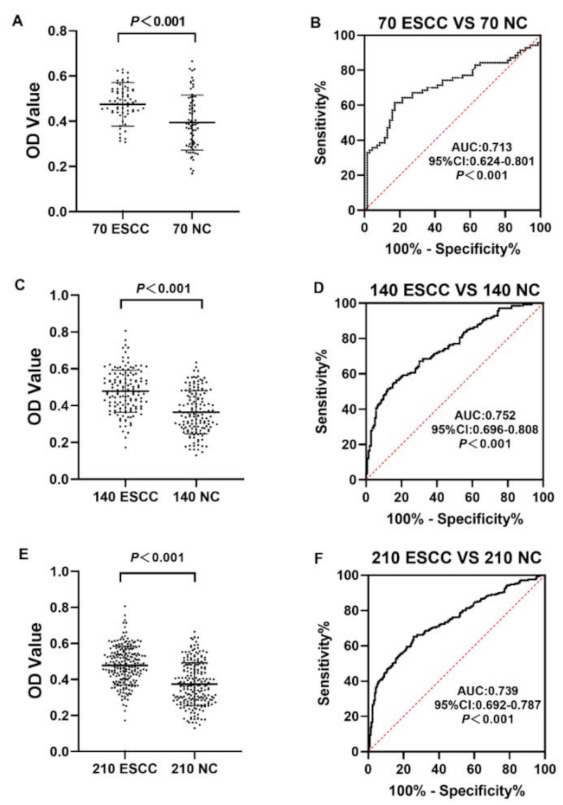
The expression level and diagnostic value of anti-CXCL8 autoantibody. The expression level of anti-CXCL8 autoantibody in the verification cohort (**A**), validation cohort (**C**) and all ESCC and all NC (**E**). The diagnostic performance of anti-CXCL8 autoantibody in the verification cohort (**B**), validation cohort (**D**) and all ESCC and all NC (**F**).

The ELISA results showed that serum anti-CXCL8 autoantibody expression level was notably higher in ESCC patients than in normal controls in both the verification set and validation set (*p* < 0.05) (Figure 4A,C). For the verification set, the AUC of anti-CXCL8 autoantibody was 0.713 (95%CI: 0.624–0.801) and the sensitivity and specificity of anti-CXCL8 autoantibody for ESCC detection were 35.7% and 82.9%, respectively (Figure 4B). For the validation cohort, the AUC of anti-CXCL8 autoantibody was 0.752 (95%CI: 0.696–0.808) and anti-CXCL8 autoantibody could distinguish 47.1% of ESCC patients at the specificity of 77.9% (Figure 4D). The statistical difference still existed when the verification set and validation set were combined as one group. The serum anti-CXCL8 autoantibody level in the ESCC group was, obviously, higher than that in the normal control group (*p* < 0.05) (Figure 4E); besides, the AUC value of anti-CXCL8 autoantibody was as high as 0.739 (95%CI: 0.692–0.787) and anti-CXCL8 autoantibody could distinguish 44.3% of ESCC patients at the specificity of 81.4% (Figure 4F). The detailed results are shown in Table 3.

**Figure 5:**
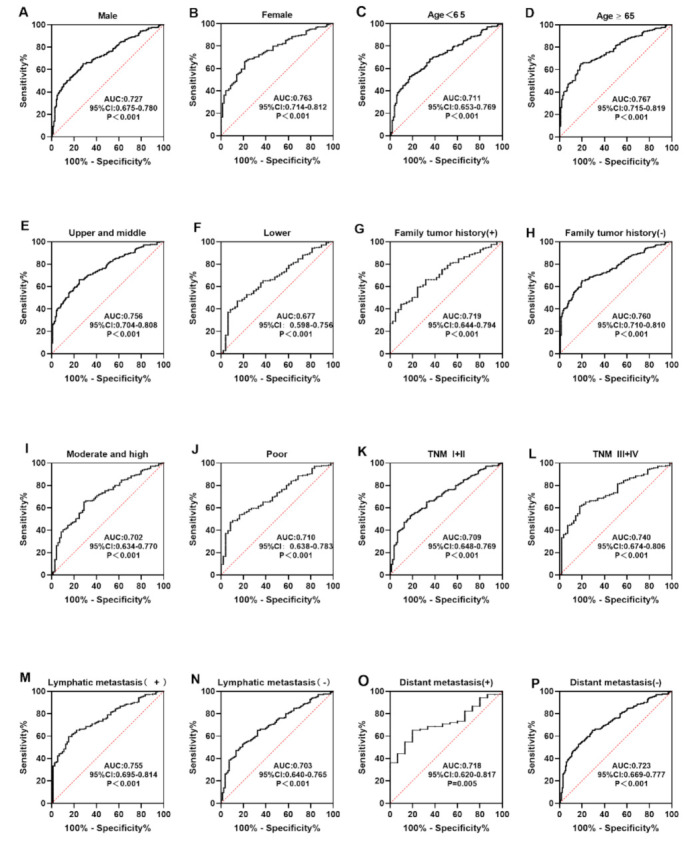
The expression of anti-CXCL8 autoantibody among subgroups of ESCC patients based on gender (**A**,**B**), age (**C**,**D**), tumor site (**E**,**F**), family tumor history (**G**,**H**), differentiation (**I**,**J**), TNM stage (**K**,**L**), lymph node metastasis (**M**,**N**), and distant metastasis (**O**,**P**).

The expression of serum anti-CXCL8 autoantibody in clinical subgroups divided by gender, age, tumor site, family tumor history, differentiation, TNM stage, lymphatic metastasis, and distance metastasis was further studied. In every subgroup, anti-CXCL8 autoantibody could significantly differentiate ESCC patients from healthy controls (*p* < 0.05) (Figure 5A–P). The AUC values of clinical subgroups ranged from 0.677 to 0.767. ESCC patients who were 65 years old or older had the highest AUC value, of 0.767 (Figure 5D), while ESCC patients with a tumor located in the lower thorax site had the lowest AUC value, of 0.677 (Figure 5F). The DeLong test showed no significant difference in AUC values among clinical subgroups (*p* > 0.05). In all clinical subgroups, there was no statistically difference in the positive frequencies of anti-CXCL8 autoantibody (*p* > 0.05) (Table 4).

## 4. Discussion

At present, the treatment level of ESCC has been significantly improved [18]. The prognosis of ESCC patients has considerably improved due to neoadjuvant therapy combined with surgery [19]. However, due to the lack of obvious early clinical symptoms and diagnostic reliable biomarkers for ESCC, many patients are still unable to obtain prompt therapy. According to numerous studies, protein biomarkers including carcino embryonic antigen (CEA), cytokeratin 19 fragment 21-1 (CYFRA21-1), and squa mous cell carcinoma antigen (SCC-Ag) can help diagnose ESCC, but they are insufficiently sensitive and specific to offer a meaningful diagnosis for ESCC [20,21]; therefore, new reliable biomarkers for ESCC are still needed.

In this study, a total of 715 genes were considered as DEGs; then, to explore potential biological functions, GO and KEGG analyses were performed. The results of GO analysis indicated that the up-regulated DEGs were significantly associated with multicellular organism development, extracellular space, and signaling receptor binding. Multicellular organism development is a complex process, and several studies have shown that the disruption of established molecular networks can drive tumor growth and many of the characteristics of cancer during multicellular organism development [22,23]. Changes in volume, shape, and composition of extracellular space play an important role in influencing tumor biological behavior. For example, in gliomas, there is a significant increase in extracellular space volume, which is also related to the malignancy of gliomas [24]. Abnormal expression and activity of signal receptors are often associated with tumorigenesis [25]. KEGG pathway analysis revealed that the DEGs were primarily related to the cytokine–cytokine receptor-interaction pathway. Cytokines influence a variety of cellular activities through their specific receptors, and they play important roles in immunity, inflammation, repair, tissue homeostasis, and hematopoiesis. Dysregulated cytokines can stimulate angiogenesis and induce immune response and immune tolerance, thereby changing the tumor microenvironment to promoting tumor pro gression [26].

In this study, through integrated bioinformatics analysis, CXCL8 was identified as the key gene for ESCC and the CXCL8 mRNA level in the ESCC group was significantly higher than in the normal control group. CXCL8 is a pro-inflammatory chemokine, also known as interleukin 8 (IL-8). CXCL8 binds to CXCr1/2 to induce inflammatory responses and new blood vessel formation, and it modulates immune responses [27]; also, CXCL8 can induce tumorigenesis by participating in the mechanism of apoptosis resistance, thereby inducing tumorigenesis [28], and it can regulate tumor angiogenesis by pro mot ing the production of matrix metalloproteinases as well as the survival and proliferation of endothelial cells [29,30]. Moreover, epithelial mesenchymal transformation (EMT) can alter the tumor microenvironment, leading to tumor-cell migration, proliferation, and immune escape, and CXCL8 can activate a variety of signaling pathways, thereby affecting EMT-related transcription factors and facilitating the formation of EMT [31]. Multiple studies have suggested that CXCL8 can induce PD-L1 + macrophages to promote the immunosuppressive microenvironment in gastric cancer [32]. CXCL8 is the recurrence marker of acute myeloid leukemia (AML) [33]. Furthermore, CXCL8 is extensively expressed in colorectal cancer, and promotes proliferation, migration, invasion, and angiogenesis of colorectal cancer cells [34]. In hepatocellular carcinoma and osteosarcoma, CXCL8 may enhance cancer-cell invasion via the PI3K/Akt signaling pathway [35,36]. These findings suggest that CXCL8 primarily acts as an oncogene, promoting tumor progression. CXCL8 phosphorylated Akt and ERK1/2 via CXCR1 and CXCR2 in vitro to promote migration and invasion of ESCC cell lines. The high expression level of CXCL8 in ESCC cancer cells is closely related to the poor prognosis of lymph node metastasis [37]. The number of CXCL8-positive tumor cells in ESCC tumor tissues is significantly increased compared with matched adjacent tissues. The expression of CXCL8 in ESCC tumor tissues is positively correlated with tumor progression and poor survival. CXCL8 secreted by primary ESCC cells can inhibit the function of natural killer cells (NK cells) through the STAT3 pathway, leading to tumor immune escape; therefore, immune-boosting therapeutic strategies targeting CXCL8 may benefit ESCC patients [38]. These studies suggest that CXCL8 plays an important role in the development of ESCC and may become a new therapeutic target for ESCC.

For various cancers, serum CXCL8 is a potential biomarker, such as for gastric cancer [39], pancreatic cancer [40], colorectal cancer [41], and non-small-cell lung cancer [42]. However, autoantibodies have advantages over other potential markers (including TAA itself) in serum persistence and stability in cancer patients [43]. Therefore, in our study, we matched patient samples with normal controls for age and sex, and designed a two-stage experiment with the verification cohort and the validation cohort to explore whether anti-CXCL8 autoantibody could be used as a diagnostic indicator for ESCC. This study was the first to explore the diagnostic value of anti-CXCL8 autoantibody for ESCC detection. According to our results, anti-CXCL8 autoantibody levels of ESCC patients were significantly higher than those of the normal control group. Anti-CXCL8 autoantibody could distinguish ESCC from healthy controls, with an AUC of 0.713 and 0.751 in the verification cohort and the validation cohort, respectively. These results indicated that anti-CXCL8 autoantibody have great potential for clinical application as a serum tumor marker for ESCC. However, there are limitations to our study. This study is a case-control study and only a preliminary exploration of the diagnostic value of anti-CXCL8 autoantibody in ESCC, which needs to be further verified in prospective studies to confirm the diagnostic value of anti-CXCL8 autoantibody in ESCC detection based on a large sample size.

## 5. Conclusions

In summary, based on the bioinformatics analysis and experimental verification presented in this study, we concluded from these results that anti-CXCL8 autoantibody could be considered a potential diagnostic marker for ESCC. This study is a case-control study, and the diagnostic value of anti-CXCL8 autoantibody in ESCC needs to be further verified in future prospective studies.

## Figures and Tables

**Table 1:** Characteristics of study participants.

Variables	Verification Phase		Validation Phase	
	(n = 140)		(n = 280)	
	ESCC (n = 70)	NC (n = 70)	ESCC (n = 140)	NC (n = 140)
Gender				
Male, n (%)	40 (57.1)	40 (57.1)	99 (70.7)	98 (70.0)
Female, n (%) Age, years	30 (42.9)	30 (42.9)	41 (29.3)	42 (30.0)
Mean age ± SD	64.28 ± 8.23	64.74 ± 8.26	63.64 ± 8.64	64.31 ± 8.75
Age range	45–88	45–84	41–87	41–88
Tumor site, n (%)				
Upper thorax	1 (1.4)		25 (17.9)	
Middle thorax	19 (27.2)		72 (51.4)	
Lower thorax	8 (11.4)		40 (28.6)	
Unknown	42 (60.0)		3 (2.1)	
Family tumor history, n (%)				
Yes	12 (17.1)		29 (20.7)	
No	56 (80.0)		79 (56.4)	
Unknown	2 (2.9)		32 (22.9)	
Histological grade, n (%)				
High	3 (4.3)		3 (2.1)	
Medium	16 (22.8)		46 (32.9)	
Low	8 (11.4)		41 (29.3)	
Unknown	43 (61.5)		50 (35.7)	
TNM stage, n (%)				
I	8 (11.4)		45 (32.2)	
II	5 (7.1)		31 (22.1)	
III	12 (17.1)		30 (21.4)	
IV	6 (8.6)		8 (5.7)	
Unknown	39 (55.7)		26 (18.6)	
Lymph node metastasis, n (%)				
Positive	18 (25.8)		54 (38.6)	
Negative	12 (17.1)		71 (50.7)	
Unknown	40 (57.1)		15 (10.7)	
Distant metastasis, n (%)				
Yes	6 (8.6)		9 (6.4)	
No	25 (35.7)		105 (75.0)	
Unknown	39 (55.7)		26 (18.6)	

Abbreviations: ESCC, esophageal squamous cell carcinoma; and NC, normal control.

**Table 2:** Functional roles of 10 hub genes.

Gene Symbol	Full Name	Degree	Function
CXCL8	C-X-C motif chemokine ligand 8	86	CXCL8 is a chemotactic factor and participates with inflammatory responses and neovascularization, and regulates immune response.
MMP9	matrix metallopeptidase 9	82	MMP9 is involved in the breakdown of extracellular matrix in normal physiological processes.
IL17A	interleukin 17A	57	IL17A mediated downstream pathways induce the production of inflammatory molecules, chemokines, and antimicrobial peptides.
SPP1	secreted phosphoprotein 1	57	The protein encoded by this gene is involved in the attachment of osteoclasts to the mineralized bone matrix. The encoded protein is secreted and binds hydroxyapatite with high affinity.
KNG1	kininogen 1	55	KNG1 is involved in signaling receptor binding and cysteine-type endopeptidase inhibitor activity.
CXCL1	C-X-C motif chemokine ligand 1	53	CXCL1 is associated with the growth and progression of certain tumors.
CXCL10	C-X-C motif chemokine ligand 10	51	Pro-inflammatory cytokine is involved in a wide variety of processes, such as chemotaxis and differentiation.
CRP	C-reactive protein	50	This gene is involved in complement activation and amplification via communication with complement initiation pattern recognition molecules.
CSF2	colony stimulating factor 2	48	CSF2 controls the production, differentiation, and function of granulocytes and macrophages.
CCL20	C-C motif chemokine ligand 20	47	CCL20 is involved in immunoregulatory and inflammatory processes.

**Table 3:** The diagnostic value of anti-CXCL8 autoantibody for ESCC.

Cohorts	AUC	95%CI	Se (%)	Sp (%)	YI	+LR	−LR	PPV (%)	NPV (%)	Accuracy
Verification	0.713	0.624–0.801	35.7	82.9	0.186	2.088	0.776	67.6	56.3	0.593
Validation	0.751	0.696–0.808	47.1	77.9	0.250	2.131	0.679	68.0	59.6	0.621
Total	0.739	0.692–0.787	44.3	81.4	0.257	2.420	0.684	70.5	59.4	0.628

Abbreviations: AUC, area under the curve; CI, confidence interval; Se, sensitivity; Sp, specificity; YI, Youden Index; +LR, positive likelihood ratio; −LR, negative likelihood ratio; FPR, false positive rate; FNR, false negative rate; PPV, positive predictive value; and NPV, negative predictive value.

**Table 4:** The positive frequencies of anti-CXCL8 autoantibody in subgroups.

Variables	Number	Frequency (%)	*p*
Gender			
Male	139	41.7	0.296
Female	71	49.3	
Age range (years)			
<65	104	38.5	0.092
≥65	106	50.0	
Family tumor history			
Yes	41	43.9	0.885
No	135	45.2	
Tumor site			
Upper and middle	117	48.7	0.119
Lower	48	35.4	
Differentiation			
Moderate and high	69	39.1	0.961
Poor	48	39.6	
TNM stage			
I–II	89	38.2	0.234
III–IV	56	48.2	
Lymphatic metastasis			
Positive	66	51.5	0.276
Negative	89	42.7	
Distant metastasis			
Yes	15	33.3	0.436
No	130	43.9	

## Data Availability

The analyzed data sets generated during the study are available from the corresponding author on reasonable request for noncommercial use.

## References

[1] Sung H., Ferlay J., Siegel R.L., Laversanne M., Soerjomataram I., Jemal A., Bray F. (2021). Global Cancer Statistics 2020: GLOBOCAN Estimates of Incidence and Mortality Worldwide for 36 Cancers in 185 Countries. CA Cancer J. Clin..

[2] Smyth E.C., Lagergren J., Fitzgerald R.C., Lordick F., Shah M.A., Lagergren P., Cunningham D. (2017). Oesophageal cancer. Nat. Rev. Dis. Primers.

[3] Pennathur A., Gibson M.K., Jobe B.A., Luketich J.D. (2013). Oesophageal carcinoma. Lancet.

[4] Wang G.Q., Jiao G.G., Chang F.B., Fang W.H., Song J.X., Lu N., Lin D.M., Xie Y.Q., Yang L. (2004). Long-term results of operation for 420 patients with early squamous cell esophageal carcinoma discovered by screening. Ann. Thorac. Surg..

[5] Bird-Lieberman E.L., Fitzgerald R.C. (2009). Early diagnosis of oesophageal cancer. Br. J. Cancer.

[6] Lao-Sirieix P., Fitzgerald R.C. (2012). Screening for oesophageal cancer. Nat. Rev. Clin. Oncol..

[7] Liang H., Fan J.H., Qiao Y.L. (2017). Epidemiology, etiology, and prevention of esophageal squamous cell carcinoma in China. Cancer Biol. Med..

[8] Codipilly D.C., Qin Y., Dawsey S.M., Kisiel J., Topazian M., Ahlquist D., Iyer P.G. (2018). Screening for esophageal squamous cell carcinoma: Recent advances. Gastrointest. Endosc..

[9] Tan E.M., Zhang J. (2008). Autoantibodies to tumor-associated antigens: Reporters from the immune system. Immunol. Rev..

[10] Heo C.K., Bahk Y.Y., Cho E.W. (2012). Tumor-associated autoantibodies as diagnostic and prognostic biomarkers. BMB Rep..

[11] Wang M., Liu F., Pan Y., Xu R., Li F., Liu A., Yang H., Duan L., Shen L., Wu Q. (2021). Tumor-associated autoantibodies in ESCC screening: Detecting prevalent early-stage malignancy or predicting future cancer risk?. EBioMedicine.

[12] Xu Y.W., Peng Y.H., Chen B., Wu Z.Y., Wu J.Y., Shen J.H., Zheng C.P., Wang S.H., Guo H.P., Li E.M. (2014). Autoantibodies as potential biomarkers for the early detection of esophageal squamous cell carcinoma. Am. J. Gastroenterol..

[13] Li C.Y., Zhang W.W., Xiang J.L., Wang X.H., Li J., Wang J.L. (2019). Identification of microRNAs as novel biomarkers for esophageal squamous cell carcinoma: A study based on The Cancer Genome Atlas (TCGA) and bioinformatics. Chin. Med. J..

[14] Bhushan A., Singh A., Kapur S., Borthakar B.B., Sharma J., Rai A.K., Kataki A.C., Saxena S. (2017). Identification and Validation of Fibroblast Growth Factor 12 Gene as a Novel Potential Biomarker in Esophageal Cancer Using Cancer Genomic Datasets. OMICS.

[15] Szklarczyk D., Morris J.H., Cook H., Kuhn M., Wyder S., Simonovic M., Santos A., Doncheva N.T., Roth A., Bork P. (2017). The STRING database in 2017: Quality-controlled protein-protein association networks, made broadly accessible. Nucleic Acids Res..

[16] Chen Z., Shen Z., Zhang Z., Zhao D., Xu L., Zhang L. (2021). RNA-Associated Co-expression Network Identifies Novel Biomarkers for Digestive System Cancer. Front. Genet..

[17] Zhang H.F., Qin J.J., Ren P.F., Shi J.X., Xia J.F., Ye H., Wang P., Song C.H., Wang K.J., Zhang J.Y. (2016). A panel of autoantibodies against multiple tumor-associated antigens in the immunodiagnosis of esophageal squamous cell cancer. Cancer Immunol. Immunother..

[18] Ohashi S., Miyamoto S., Kikuchi O., Goto T., Amanuma Y., Muto M. (2015). Recent Advances From Basic and Clinical Studies of Esophageal Squamous Cell Carcinoma. Gastroenterology.

[19] Lin H.N., Chen L.Q., Shang Q.X., Yuan Y., Yang Y.S. (2020). A meta-analysis on surgery with or without postoperative radiotherapy to treat squamous cell esophageal carcinoma. Int. J. Surg..

[20] Zheng Q., Zhang L., Tu M., Yin X., Cai L., Zhang S., Yu L., Pan X., Huang Y. (2021). Development of a panel of autoantibody against NSG1 with CEA, CYFRA21-1, and SCC-Ag for the diagnosis of esophageal squamous cell carcinoma. Clin. Chim. Acta.

[21] Wang X.B., Jiang X.R., Yu X.Y., Wang L., He S., Feng F.Y., Guo L.P., Jiang W., Lu S.H. (2014). Macrophage inhibitory factor 1 acts as a potential biomarker in patients with esophageal squamous cell carcinoma and is a target for antibody-based therapy. Cancer Sci..

[22] Trigos A.S., Pearson R.B., Papenfuss A.T., Goode D.L. (2018). How the evolution of multicellularity set the stage for cancer. Br. J. Cancer.

[23] Trigos A.S., Pearson R.B., Papenfuss A.T., Goode D.L. (2017). Altered interactions between unicellular and multicellular genes drive hallmarks of transformation in a diverse range of solid tumors. Proc. Natl. Acad. Sci. USA.

[24] Zamecnik J. (2005). The extracellular space and matrix of gliomas. Acta Neuropathol..

[25] O’Hayre M., Vazquez-Prado J., Kufareva I., Stawiski E.W., Handel T.M., Seshagiri S., Gutkind J.S. (2013). The emerging mutational landscape of G proteins and G-protein-coupled receptors in cancer. Nat. Rev. Cancer.

[26] Diakowska D. (2013). Cytokines association with clinical and pathological changes in esophageal squamous cell carcinoma. Dis. Markers.

[27] Ha H., Debnath B., Neamati N. (2017). Role of the CXCL8-CXCR1/2 Axis in Cancer and Inflammatory Diseases. Theranostics.

[28] Kumar S., O’Malley J., Chaudhary A.K., Inigo J.R., Yadav N., Kumar R., Chandra D. (2019). Hsp60 and IL-8 axis promotes apoptosis resistance in cancer. Br. J. Cancer.

[29] Li A., Dubey S., Varney M.L., Dave B.J., Singh R.K. (2003). IL-8 directly enhanced endothelial cell survival, proliferation, and matrix metalloproteinases production and regulated angiogenesis. J. Immunol..

[30] Kumar A., Cherukumilli M., Mahmoudpour S.H., Brand K., Bandapalli O.R. (2018). ShRNA-mediated knock-down of CXCL8 inhibits tumor growth in colorectal liver metastasis. Biochem. Biophys. Res. Commun..

[31] Long X., Ye Y., Zhang L., Liu P., Yu W., Wei F., Ren X., Yu J. (2016). IL-8, a novel messenger to cross-link inflammation and tumor EMT via autocrine and paracrine pathways (Review). Int. J. Oncol..

[32] Lin C., He H., Liu H., Li R., Chen Y., Qi Y., Jiang Q., Chen L., Zhang P., Zhang H. (2019). Tumour-associated macrophages-derived CXCL8 determines immune evasion through autonomous PD-L1 expression in gastric cancer. Gut.

[33] Li Y., Cheng J., Li Y., Jiang Y., Ma J., Li Q., Pang T. (2018). CXCL8 is associated with the recurrence of patients with acute myeloid leukemia and cell proliferation in leukemia cell lines. Biochem. Biophys. Res. Commun..

[34] Yu L., Yang X., Xu C., Sun J., Fang Z., Pan H., Han W. (2020). Comprehensive analysis of the expression and prognostic value of CXC chemokines in colorectal cancer. Int. Immunopharmacol..

[35] Sun F., Wang J., Sun Q., Li F., Gao H., Xu L., Zhang J., Sun X., Tian Y., Zhao Q. (2019). Interleukin-8 promotes integrin beta3 upregulation and cell invasion through PI3K/Akt pathway in hepatocellular carcinoma. J. Exp. Clin. Cancer Res..

[36] Jiang H., Wang X., Miao W., Wang B., Qiu Y. (2017). CXCL8 promotes the invasion of human osteosarcoma cells by regulation of PI3K/Akt signaling pathway. APMIS.

[37] Hosono M., Koma Y.I., Takase N., Urakawa N., Higashino N., Suemune K., Kodaira H., Nishio M., Shigeoka M., Kakeji Y. (2017). CXCL8 derived from tumor-associated macrophages and esophageal squamous cell carcinomas contributes to tumor progression by promoting migration and invasion of cancer cells. Oncotarget.

[38] Wu J., Gao F.X., Wang C., Qin M., Han F., Xu T., Hu Z., Long Y., He X.M., Deng X. (2019). IL-6 and IL-8 secreted by tumour cells impair the function of NK cells via the STAT3 pathway in oesophageal squamous cell carcinoma. J. Exp. Clin. Cancer Res..

[39] Pawluczuk E., Lukaszewicz-Zajac M., Gryko M., Kulczynska-Przybik A., Mroczko B. (2021). Serum CXCL8 and Its Specific Receptor (CXCR2) in Gastric Cancer. Cancers.

[40] Litman-Zawadzka A., Łukaszewicz-Zając M., Gryko M., Kulczyńska-Przybik A., Mroczko B. (2018). Serum chemokine CXCL-8 as a better biomarker for diagnosis and prediction of pancreatic cancer than its specific receptor CXCR-2, CRP and classical tumor markers (CA 19-9 and CEA). Pol. Arch. Intern. Med..

[41] Paczek S., Lukaszewicz-Zajac M., Gryko M., Mroczko P., Kulczynska-Przybik A., Mroczko B. (2020). CXCL-8 in Preoperative Colorectal Cancer Patients: Significance for Diagnosis and Cancer Progression. Int. J. Mol. Sci..

[42] Cai D., Xu Y., Ding R., Qiu K., Zhang R., Wang H., Huang L., Xie X., Yan H., Deng Y. (2020). Extensive serum biomarker analysis in patients with non-small-cell lung carcinoma. Cytokine.

[43] Anderson K.S., LaBaer J. (2005). The sentinel within: Exploiting the immune system for cancer biomarkers. J. Proteome Res..

